# Threshold effect of government subsidy, corporate social responsibility and brand value using the data of China’s top 500 most valuable brands

**DOI:** 10.1371/journal.pone.0251927

**Published:** 2021-05-25

**Authors:** Yongzhi Qi, Yuchen Chai, Yifan Jiang

**Affiliations:** School of Business Administration, Shanxi University of Finance and Economics, Taiyuan, China; Taiyuan University of Science and Technology, CHINA

## Abstract

An increasing number of firms have begun to attach importance to corporate social responsibility (CSR) to obtain sustainable strategic advantages in the competitive market. On the basis of nonlinear perspective, panel data of A-share listed companies in the ranking list of China’s Top 500 Most Valuable Brands in 2012–2018 and Hansen panel threshold regression technology were adopted. With government subsidy and CSR being threshold variables, the internal mechanism about the influence of government subsidy and CSR on brand value was explored. Results show that the following. (1) CSR has a significantly inverted U-type threshold effect on brand value. (2) Government subsidy facilitates CSR with diminishing marginal utility. (3) When a difference exists in the strength of government subsidy, the influence of CSR on brand value presents a significant N-type law. Furthermore, threshold regression method was used to innovatively explore the complex nonlinear relationship among government subsidy, CSR, and brand value. This relationship has a significantly practical significance for listed firms for weighing the business decisions regarding the input of CSR and brand value, as well as subsidy policies for enterprises by the government.

## Introduction

According to Mundell, winner of Nobel Economics Prize, brand is the language for global communication and the name card of a nation [1]. Under a fierce economic environment, brand can create stronger product premium and competitive edges as an important symbol of enterprises [2]. At present, brand value has become an intangible asset being fiercely pursued by enterprises. From the perspective of enterprise marketing management, fulfilling social responsibility can yield good reputation and obtain identification of stakeholders, such as customers for enterprises, thereby generating brand effect [3] and improving sustainable development of enterprises [4, 5]. However, major differences still exist in the relationship between corporate social responsibility (CSR) and brand value in academic circle. One of the advantages of performing social responsibility is winning public recognition, enhancing popularity and reputation of brand [6], and promoting brand value [7]. However, the fulfilment of CSR will crowd out the resources for building a brand [8] and increase the risk of being perceived as a hypocrisy behavior and commercial grandstanding in pursuit of higher benefits [9], thereby restraining the promotion of brand value or even causing the decline in brand value. Moreover, relevance exists among CSR, social goals of the government, and government performance assessment indicators. Efficient government subsidy policy can create a good institutional environment for enterprises and encourage them to actively participate in social responsibility activities [10] and then enhance their brand value. CSR also becomes feedback after obtaining government subsidies, including increasing employment and tax contribution. Therefore, the effect of government subsidy on the performance of CSR cannot be ignored [11]. Few scholars have explored the influence of government subsidy on CSR, as well as its further influence on the relationship between CSR and brand value.

On the basis of the above analysis, enterprises among A-share listed companies ranking on China’s Top 500 Most Valuable Brands for seven years issued by the World Brand Lab in 2012–2018 are selected, and 770 samples are obtained for this study. On the basis of nonlinear perspective, this study aims to reveal the internal mechanism of CSR on brand value under the situation of government subsidy. In this manner, this study can provide guidance for enterprises to perform social responsibility decisions, offering theoretical reference and empirical evidence to the government for perfecting decisions about subsidies. The contributions of this study mainly include the following. (1) On the basis of the existing relationship between CSR and brand value, the variable of government subsidy is introduced, and mutual relation between them is integrated, thereby expanding the theoretical perspective of existing research. (2) From a nonlinear perspective, a panel threshold data model is adopted to study the relationship among government subsidy, CSR, and brand value, thereby effectively distinguishing boundary conditions of the relationship among the three variables and compensate for the shortcomings of the current research, mainly focusing on linear relationship.

The remainder of this paper is organized as follows. The second section provides a literature review of theoretical and empirical studies. The methodology is elaborated in the third section, and the results analysis and discussions are presented in the fourth section. The last section summarizes the main findings of the study and concludes with the managerial implications for further research.

## Literature review

### CSR and brand value

The current research holds two opposite views about the relationship between CSR and brand value. Some scholars consider that performing social responsibility is beneficial to promote the brand value of enterprises. First, Chinese enterprises still treat the performance of social responsibility as a type of “advertisement” marketing behavior [12], considering that association produced by CSR can deliver altruistic value of enterprises in a warmhearted manner, that is, satisfying customer and social demands, which is beneficial in obtaining brand reputation [13] and then enhancing brand value [14, 15]. Second, brand fulfilling social responsibility is favored by consumers, which can establish a strong emotional connection with consumers [16] and bring high market share to enterprises [17], which can positively influence brand value. Furthermore, the source of brand value is its future profitability, and it is the willingness and motivation of consumers to continuously pay for brand beyond product or service premium [18]. According to Demetriou et al., corporate measures supporting and facilitating social prosperity may increase additional value of current products/services [19]. Lerro et al. indicate that consumers usually tend to purchase enterprise products with good public praise of social responsibility [20]. CSR is not only helpful for enhancing the purchasing intention of consumers and improving public evaluation on enterprises/brands [21] but also beneficial for brand extension [22]. The research results of Lai et al. also show that CSR activities have a positive influence on brand performance [23]. In conclusion, CSR can win recognition from the public and stakeholders, enhance popularity and brand reputation [24, 25], and promote brand competitiveness on the market, thereby producing positive influence on brand value [26].

Other scholars think that the input of CSR hinders the promotion of brand value. At first, in the process of achieving improved performance, enterprises have extensive contact with stakeholders, which makes them pay attention to CSR. At the same time, they expect pressure in various ways, which makes enterprises bear the social responsibility of paying high costs, which crowd out the resources needed for brand value building [27, 28] and lead to the decline of brand value. Furthermore, if social responsibility behaviors of some enterprises are considered by the public negatively, such as a commercial show or moral hypocrisy, then they cannot bring a positive reputation asset to enterprises and even corrode corporate reputation [29]. If the field of CSR is only related with its business, stakeholders will think that CSR aims to eliminate negative externalities of its main business activity or gain high profits, which will bring negative effect to the brand [30]. By analyzing the panel data of A-share listed companies, Li et al. find that CSR has a negative inhibition effect on brand value [31]. Therefore, blind performance of social responsibility can lead to irrational resource allocation, and false social responsibility will damage brand value [32].

### Government subsidy and CSR

Research conclusions about the promotion or inhibition effect of government subsidy on CSR by domestic and foreign scholars differ. Some scholars consider that government subsidy is a nonmarket means of the government to overcome the externality of the environment and facilitate environmental improvement, which promotes CSR inputs [33]. First, government subsidies provide a capital source for enterprises, relieve financing constraints, and improve corporate operation state [34]; thus, enterprises can undertake social responsibility. The research by Zeng et al. shows that government subsidies can expand the cash flow of enterprises, increase external financing scale, reduce financing costs, and effectively solve the problem of financing constraints during production and operation process [35]. After obtaining government subsidies, enterprises can deliver good development prospect to others and attract attention of external investment organizations, such as banks, thereby increasing the probability of obtaining short- and long-term bank loans and investment organizations [36, 37]. With a rich cash flow, enterprises can finish production and operation activities effectively, and they can and are willing to undertake social responsibilities. Second, obtaining political resources or lowering political costs is a powerful motivation for enterprises to fulfill social responsibility; that is, CSR activities have become a way to consolidate government–enterprise association [38]. With government subsidies, enterprises will initiatively assume social responsibility in return to support government to realize political or social goals [39]. Donations are relatively a direct way for enterprises to perform social responsibilities. Private enterprises utilize donations to consolidate government–enterprise association [40], and money-losing enterprises with political resources tend to obtain government support through charitable donations [41]. If state-owned enterprises with tight government–enterprise association do not assume social responsibility actively, then they will undertake higher political costs and may lose government support, such as preferential policy and resource subsidy [42]. To develop and maintain government–enterprise relationship and acquire continuous support from the government, enterprises are motived to repay the government and society through social responsibilities. Therefore, enterprises with government subsidies will fulfill social responsibility actively. On the one hand, government subsidies improve enterprise operation state, and enterprises, in turn, can and are willing to perform social responsibilities. On the other hand, government–enterprise association is enhanced, enterprises continuously obtain government subsidy resources, and increasing political costs caused by failure to perform social responsibilities is avoided.

Other scholars claim that government subsidies hinder CSR inputs. First, abuse of government subsidy funds reduces CSR inputs. Under the situation of weak constraints, government subsidies may induce moral risks. For example, some enterprises grant executive compensation with government subsidies, making it difficult to realize the goal of government subsidies [43–45]. Possible corruption and rent-seeking behavior during the allocation process of government subsidy funds hinder efficient allocation and weaken subsidy effect [46]. Moreover, government subsidies may influence enterprise operation performance, and implementing social responsibility activities is not beneficial. Money-losing listed companies can improve their financial condition in the year with government subsidies; however, enterprises will depend on government subsidy in the long term, which is not beneficial to improve operation performance [47]. Through government subsidies, controlling shareholders increase surplus while reducing earnings quality [48], which leads to inefficiency of government subsidy [49]. With poor earnings quality, few extra resources will be inputted for social responsibility activities. Consequently, government subsidies have a negative influence on CSR. On the one hand, abuse of government subsidy reduces CSR inputs. On the other hand, government subsidies lead to dependence of enterprise operation and no idle resources are inputted for CSR.

In conclusion, regarding the relationship between CSR and brand value and between government subsidy and CSR, most studies at home and abroad tend to focus on the perspective of the linear effect between variables, and no consistent conclusion exists at present. Actually, diversity of research conclusions indicates a complex relationship between CSR and brand value and between government subsidy and CSR to some extent, and complex nonlinear relationship may exist between them. What type of complex mechanism will government subsidy produce on the effect between CSR and brand value? What type of characteristics and rules will be produced? If a threshold exists, then how will the characteristics of the threshold be manifested? These issues need to be discussed further.

## Methodology

### Model design

As per the panel threshold effect model proposed by Hansen [50], three threshold models with a single threshold value are initially constructed, and a multiple-threshold model is then gradually expanded. The three models of a single threshold value are as follows.

Model (1) is the threshold regression model, with CSR being the threshold variable and independent variable at the same time, with brand value being the dependent variable.

$$BV_{it}=\mu_{i}+\theta^{'}X_{it}+\beta_{1}CSR_{it}I(CSR_{it}\leq \gamma )+\beta_{2}CSR_{it}I(CSR_{it}>\gamma )+\varepsilon_{it}$$ (1)

Model (2) the is regression model, with government subsidy being the threshold variable and independent variable at the same time, with CSR being the dependent variable.

$$CSR_{it}=\mu_{i}+\theta^{'}X_{it}+\beta_{1}Sub_{it}I(Sub_{it}\leq \gamma )+\beta_{2}Sub_{it}I(Sub_{it}>\gamma )+\varepsilon_{it}$$ (2)

Model (3) is the threshold model, with government subsidy being the threshold variable, CSR being the independent variable, and brand value being the dependent variable.

$$BV_{it}=\mu_{i}+\theta^{'}X_{it}+\beta_{1}CSR_{it}I(Sub_{it}\leq \gamma )+\beta_{2}CSR_{it}I(Sub_{it}>\gamma )+\varepsilon_{it}$$ (3)

In the models, *i* refers to a specific company; *t* is the year of a company; corresponding coefficient vectors are expressed with *β*_*1*_, *β*_*2*_, and *θ*′; *Sub*_*it*_, *CSR*_*it*_, and *BV*_it_ refer to government subsidy, input of CSR, and brand value, respectively; and *γ* is a specific threshold value. *X*_*it*_ is a control variable, including enterprise size, financial leverage, shareholder balance, and return on total assets, which may obviously influence brand value and CSR. I(·) is an exponential function, *μ*_*i*_ represents the individual effect of the enterprise, and ε_it_ is a random disturbance term. Specific meanings and calculation methods of the variables are as follows.

Models (1)–(3) are combined as

$$Tv_{it}=\mu_{i}+\theta^{'}X_{it}+\beta_{1}Tr_{it}I(Ts_{it}\leq \gamma )+\beta_{2}Tr_{it}I(Ts_{it}>\gamma )+\varepsilon_{it}.$$ (4)

If *Tv* in Formula (4) is *BV*, then it is Models (1) and (3). If *Tv* is *CSR*, then it is Model (2). If *Tr* is *CSR*, then it is Models (1) and (3). If *Tr* is *Sub*, then it is Model (2). If *Ts* is *CSR*, then it is Model (1). If *Ts* is *Sub*, then it is Models (2) and (3).

To estimate the parameters, the interclass mean is subtracted from each observed value to eliminate the individual effect, and the model after conversion is

$$Tv_{it}^{*}=\theta^{'}X_{it}^{*}+\beta_{1}Tr_{it}^{*}I(Ts_{it}\leq \gamma )+\beta_{2}Tr_{it}^{*}I(Ts_{it}>\gamma )+\varepsilon_{it}^{*}.$$ (5)

All observed values are superimposed, and Formula (5) is expressed in matrix form, as shown as follows:

$$Tv^{*}=\theta^{'}X(\gamma )\beta +\varepsilon^{*}.$$ (6)

For a given threshold value *γ*, the least square estimation of Formula (6) is adopted, and the value of *β* is obtained as follows:

$$\hat{\beta }(\gamma )={\left(X^{*}{\left(\gamma \right)}^{'}X^{*}(\gamma )\right)}^{-1}X^{*}{\left(\gamma \right)}^{'}Tv^{*}).$$ (7)

The corresponding residual sum of squares is

$$S_{1}(\gamma )={\hat{e}}^{*}{\left(\gamma \right)}^{'}{\hat{e}}^{*}(\gamma ),$$ (8)

Where ${\hat{e}}^{*}(\gamma )=Tv^{*}-X^{*}(\gamma )\hat{\beta }(\gamma )$ is the residual vector. By using the minimum of Formula (8), the estimated value of *γ* can be obtained as

$$\hat{\gamma }=\underset{\gamma }{\arg}\;\min S_{1}(\gamma ).$$ (9)

Then, whether the threshold effect and the estimated value are equal to the true value is tested. The null hypothesis and corresponding alternative hypothesis of the threshold effect test are respectively as follows: H_0_: *β*_*1*_ *= β*_*2*_ and H_1_: *β*_*1*_ *≠ β*_*2*_. The test statistics can be expressed as follows:

$$F_{1}=\frac{S_{0}-S_{1}(\gamma )}{\hat{\sigma^{2}}}.$$ (10)

Under the condition of the null hypothesis, the threshold value cannot be identified. As a result, *F*_*1*_ statistics is an abnormal distribution. The null hypothesis of the second test is H_0_: *γ = γ*_*0*_, and the corresponding likelihood ratio statistics is

$$LR_{1}(\gamma )=\frac{SSE_{1}(\gamma )‐SSE_{1}(\hat{\gamma })}{\hat{\sigma^{2}}}.$$ (11)

Given that the distribution of the statistics is abnormal, this study refers to the formula proposed by Hansen (1999) to obtain the nonrejection region, that is, if *LR*_*1*_ (*γ*_*0*_) *≤ c*(*α*), then the null hypothesis cannot be refused, in which *c*(*α*) *= −2ln(1 − (1 − α)*^*1/2*^*)*, where *α* indicates a significant level.

A threshold exists in the above hypothesis, namely, parameter estimation and hypothesis testing process of the single-threshold panel model. The dual model is expanded into a multiple model, as shown as follows:

$$Tv_{it}=\mu_{i}+\theta^{'}X_{it}+\beta_{1}Tr_{it}I(Ts_{it}\leq \gamma_{1})+\beta_{2}Tr_{it}I(\gamma_{1}<Ts_{it}\leq \gamma_{2})+\beta_{3}Tr_{it}I(Ts_{it}>\gamma_{2})+\varepsilon_{it}.$$ (12)

First, the estimated value of known ${\hat{\gamma }}_{1}$ is hypothesized and *γ*_2_ is searched, which yields the following:

$$S_{2}^{r}(\gamma_{2})=\{\begin{array}{c} s(\hat{\gamma_{1}},\gamma_{2})if\;\hat{\gamma_{1}}<\gamma_{2}\\ s(\gamma_{2},\hat{\gamma_{1}})if\;\gamma_{2}<\hat{\gamma_{1}} \end{array}\;\mathrm{and}\;{\hat{\gamma }}_{2}^{r}=\underset{r}{\arg}\;\min S_{2}^{r}(\gamma_{2}).$$

This study shows that $\hat{\gamma_{2}^{r}}$ is gradual and effective, whereas $\hat{\gamma_{1}}$ does not show this nature. Here, $\hat{\gamma_{2}^{r}}$ can be fixed, and $\hat{\gamma_{1}}$ is searched again to obtain a consistent statistic after optimization. Parameter estimation and hypothesis testing process of the multiple-threshold model are similar to those of the single-threshold model, as shown as follows:

$$Tv_{it}=\mu_{i}+\theta^{'}X_{it}+\beta_{1}Tr_{it}I(Ts_{it}\leq \gamma_{1})+\beta_{2}Tr_{it}I(\gamma_{1}<Ts_{it}\leq \gamma_{2})+\cdots \cdots +\beta_{n}Tr_{it}I(\gamma_{n-1}<Ts_{it}\leq \gamma_{n})+\beta_{n+1}Tr_{it}I(Ts_{it}>\gamma_{n})+\varepsilon_{it}.$$ (13)

### Sample and variable selection

The selection interval of the samples in this study is the listed companies in China’s Top 500 Most Valuable Brands issued by the World Brand Lab in 2012–2018, and the samples are screened as follows. (1) Enterprises among A-share listed companies in Shanghai and Shenzhen ranking for seven years are selected. (2) Financial and insurance companies are removed, because their statement structures and main titles of account are different from those of the general industry, and their business models also differ from other types of enterprise. (3) Samples under abnormal transaction status are removed, including ST, SB, *ST, and PT share. (4) Missing value sample is removed. After screening, the cross-section data of 110 sample companies are finally obtained, with a total of 770 samples.

The explained variable is brand value (*BV*), and the natural logarithm of brand value data in China’s Top 500 Most Valuable Brands issued by the World Brand Lab is used for measurement. Government subsidy (*Sub*) and the input strength of CSR (*CSR*) are threshold variables, and *CSR* is also an independent variable. With regard to government subsidy, the natural logarithm of government subsidy amount received by enterprises is used for measurement. For the measurement of the input strength of CSR, this study refers to the method of Shen et al. to measure with social contribution per share [51]. Social contribution per share = (net profit + business tax and surcharges + donation fee + financial expense + income tax expense–pollution discharge and clearing expense + cash paid to staff and paid for staff + employee pay payable in current period − employee pay payable in prior period)/mean of total shares at the beginning and end of the period. The control variables are the following: enterprise size (*size*, natural logarithm of enterprise total assets); financial leverage (*Lev*, gross liabilities/total assets); return on total assets (Roa, net profit/total assets); shareholder balance (*Shb*, sum of ratio of the second to tenth largest shareholders/shareholding amount of the largest shareholder); shareholding ratio of board of directors (*Bsr*, total shareholding of board members/total share of company); ratio of independent directors (*Indep*, number of independent directors/total number of board members); and duality (*Dual*, *Dual* = 1, duality; *Dual* = 0, not duality).

## Results analysis and discussions

In Model (1), CSR is the threshold variable and independent variable, and brand value is the dependent variable. In Model (2), government subsidy is the threshold variable and independent variable, and CSR is the dependent variable. In Model (3), government subsidy is the threshold variable, CSR is the independent variable, and brand value is the dependent variable. The test result of the threshold effect of the three models show that the single and double thresholds of the three models pass the significance test, and the triple threshold is insignificant (Tables 1 and 2). As shown in Table 2, the threshold values of Model (1) are 0.957 and 2.479, those of Model (2) are 13.217 and 17.148, and those of Model (3) are 18.184 and 22.803. This study conducts a specific analysis of the dual threshold effect. The biased error of the estimation structure caused by important threshold factors of government subsidy and CSR is neglected.

**Table 1:** Test of threshold effect.

Model		Critical value				
		F-value	P-value	1%	5%	10%
Model (1)	Single threshold	17.840***	0.000	14.219	9.996	8.528
	Dual threshold	12.934*	0.053	19.733	13.325	10.563
	Triple threshold	0.000	0.430	0.000	0.000	0.000
Model (2)	Single threshold	36.738***	0.007	30.039	18.473	11.954
	Dual threshold	13.186***	0.003	9.865	5.250	3.993
	Triple threshold	0.000	0.163	0.000	0.000	0.000
Model (3)	Single threshold	8.687*	0.080	14.262	10.510	8.274
	Dual threshold	33.803***	0.000	23.878	13.467	11.668
	Triple threshold	-0.000	0.690	0.000	0.000	0.000

Note: (1) Critical value and P value are obtained by adopting Bootstrap repeated self-sampling for 300 times; (2) ***, **, and * represent significant correlation in the 1%, 5%, and 10% levels, respectively.

**Table 2:** Threshold value and confidence interval estimation.

Model	Item	Threshold value	95% interval estimation
Model (1)	Threshold value 1	0.957	[0.916,6.575]
	Threshold value 2	2.479	[0.248,6.303]
Model (2)	Threshold value 1	13.217	[13.217,13.505]
	Threshold value 2	17.148	[16.378,21.172]
Model (3)	Threshold value 1	18.184	[17.910,18.522]
	Threshold value 2	22.803	[20.972,23.060]

**Fig 1:**
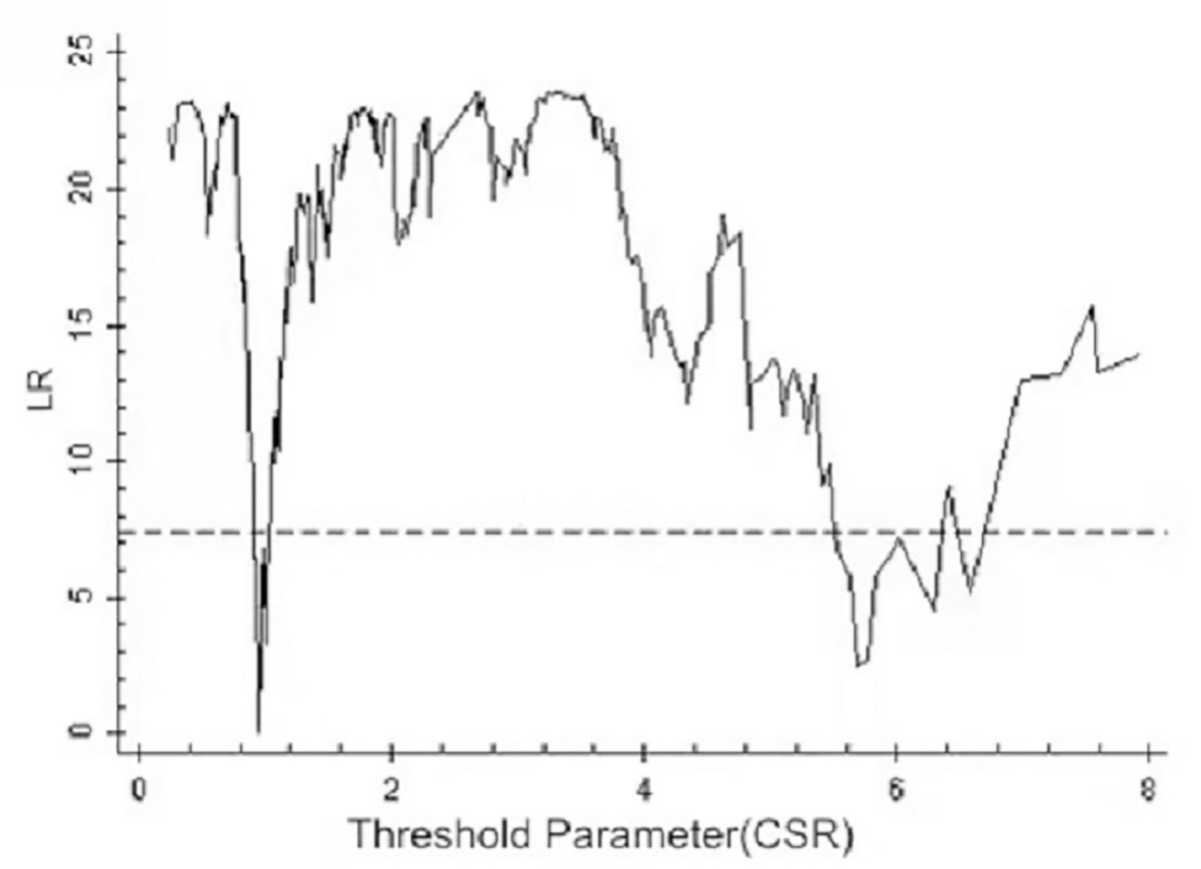
Estimated value and confidence interval of the first threshold in Model 1.

**Fig 2:**
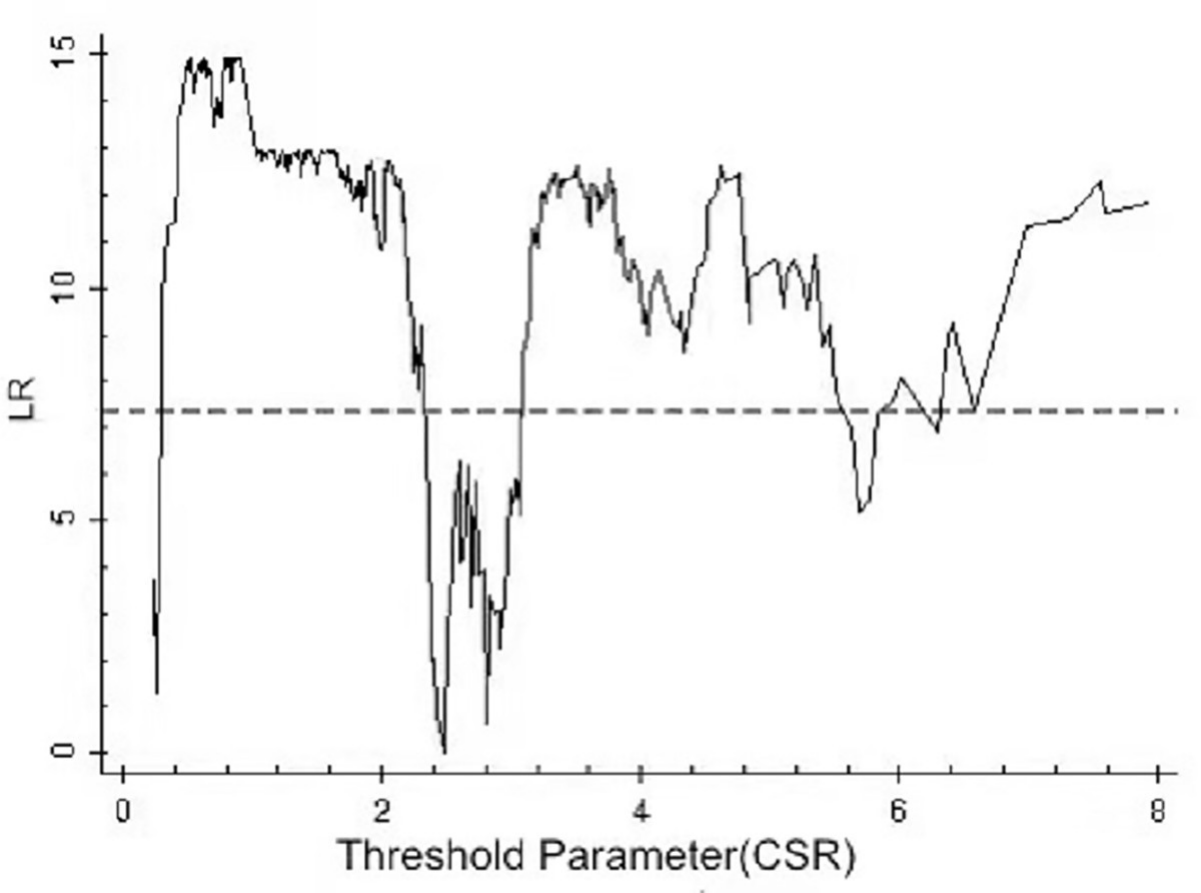
Estimated value and confidence interval of the second threshold in Model 1.

**Fig 3:**
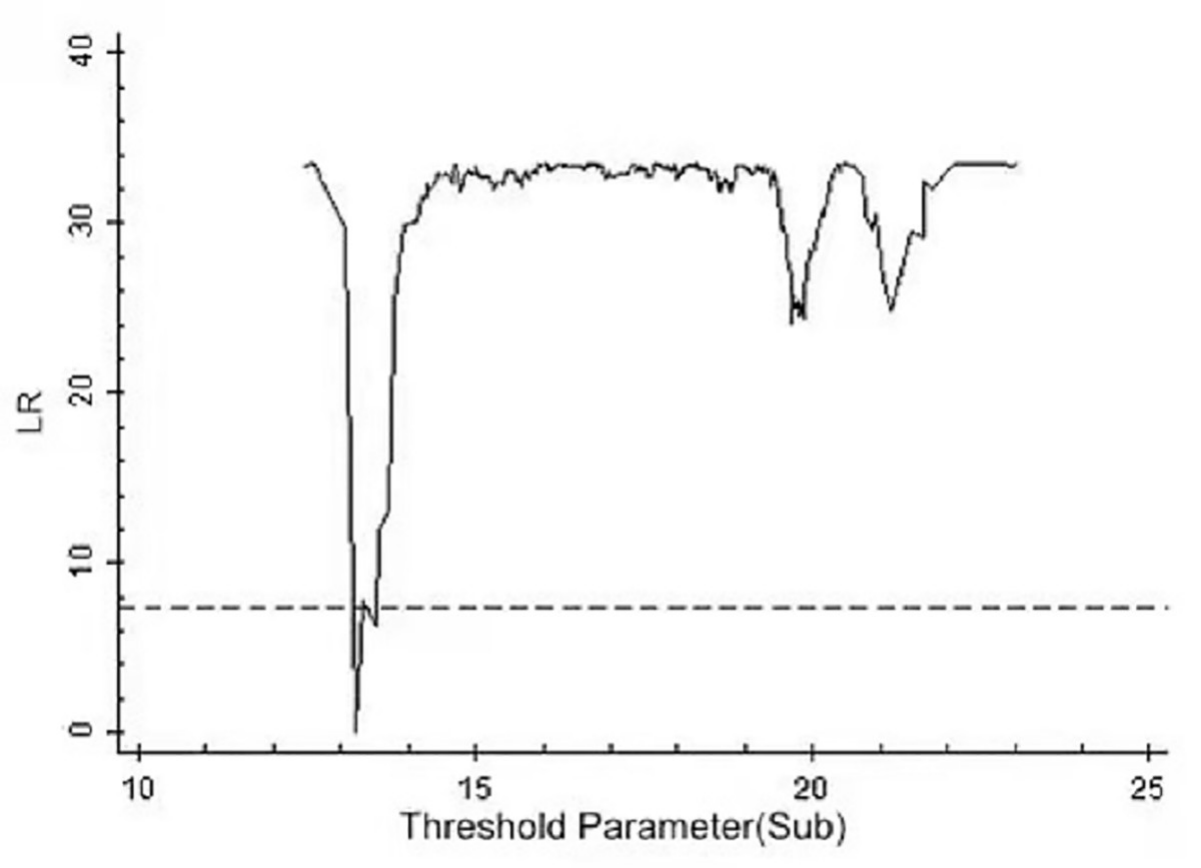
Estimated value and confidence interval of the first threshold in Model 2.

**Fig 4:**
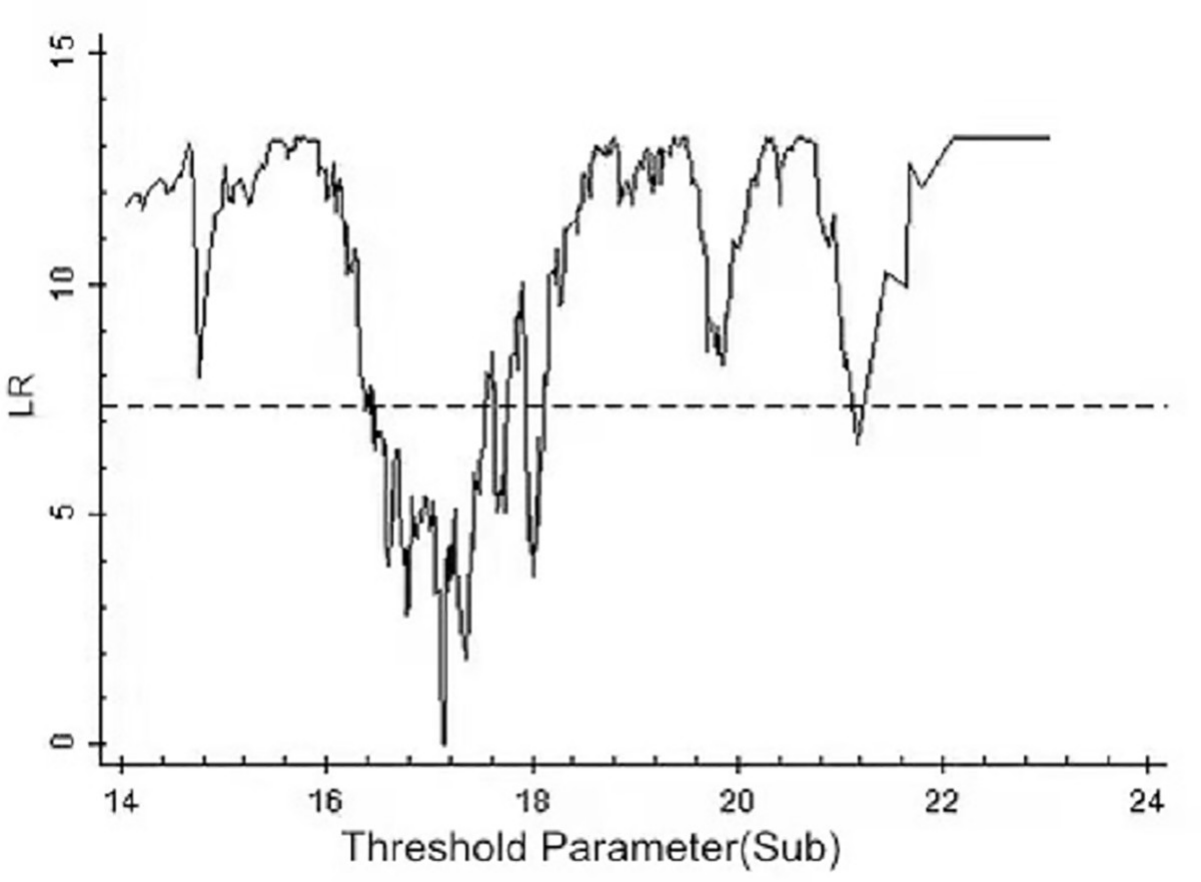
Estimated value and confidence interval of the second threshold in Model 2.

**Fig 5:**
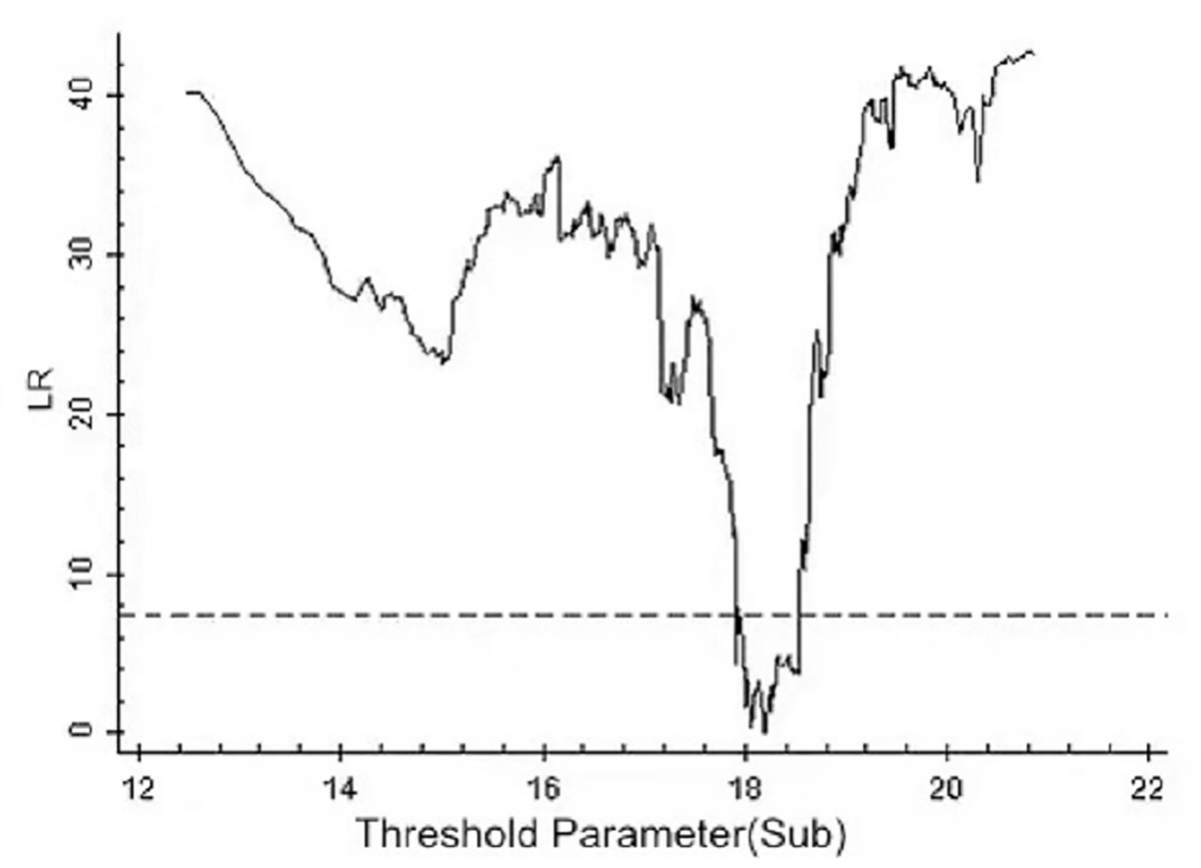
Estimated value and confidence interval of the first threshold in Model 3.

**Fig 6:**
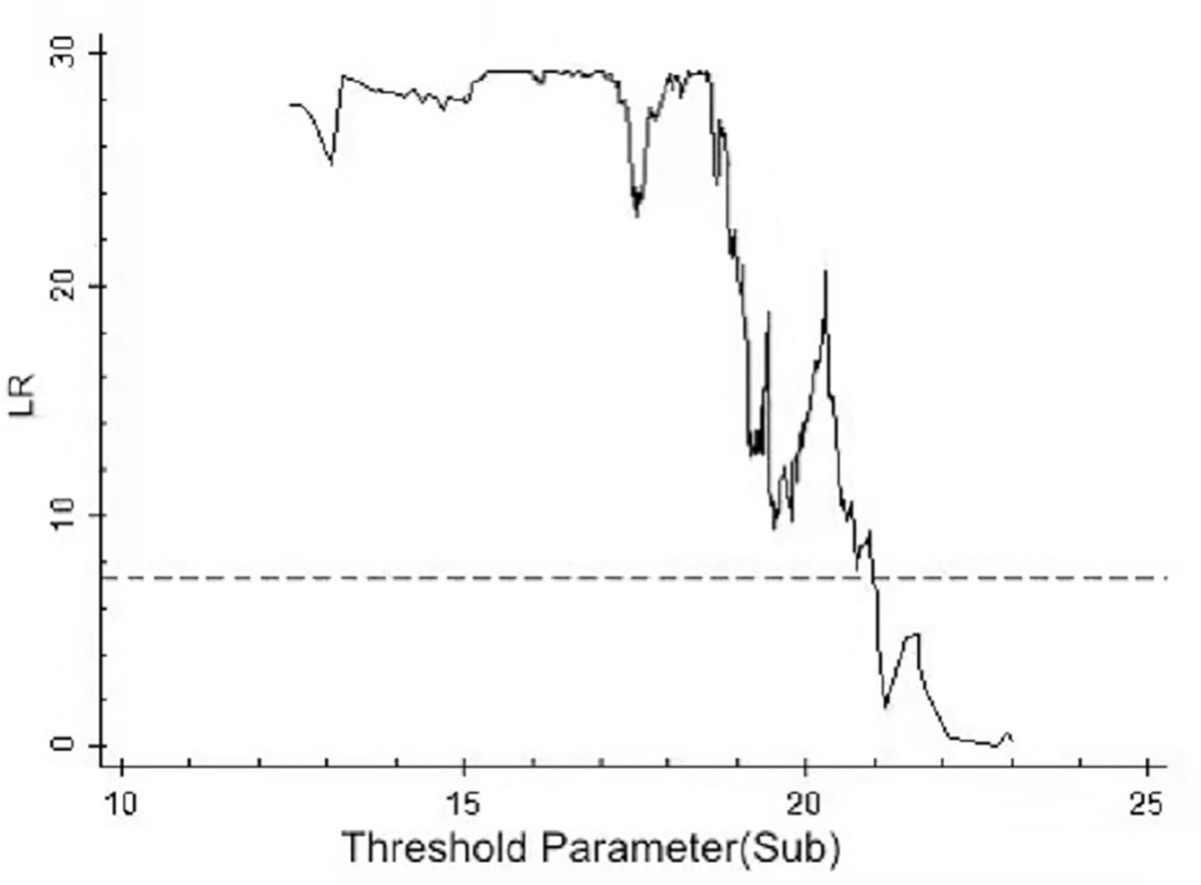
Estimated value and confidence interval of the second threshold in Model 3.

By using the above threshold test, the threshold value and 95% confidence interval of each model are illustrated as drawn graphs of the function of likelihood ratio, as shown in Figs 1–6. The likelihood ratio statistics of the three models are zero. The 95% confidence interval of the estimated value of each threshold is the interval composed by the value of γ of the critical value (corresponding to the dotted lines in Figs 1–6) under the significant level of all LR values being less than 5%. Moreover, the truth test of the estimated value of the threshold is passed. As a result, the three models can be divided into low, medium, and high types as per the two threshold values.

On the basis of the estimation of panel threshold value, the samples are divided to different intervals as per threshold value, and the threshold regression analysis is conducted. The function of the threshold effect is tested by comparing the differences of the slope coefficient within three intervals. Table 3 shows the estimation results.

**Table 3:** Parameter estimation results of dual-threshold model.

	Model (1)			Model (2)			Model (3)		
Variable	Estimated coefficient	T value	P value	Estimated coefficient	T value	P value	Estimated coefficient	T value	P value
c	1.010**	2.09	0.037	-18.277***	-11.22	0.000	0.406	0.81	0.418
lnSize	0.209***	9.55	0.000	0.738***	10.45	0.000	0.233***	10.28	0.000
Lev	-1.348***	-9.25	0.000	1.237***	2.61	0.009	-1.346***	-9.27	0.000
Roa	-2.181***	-5.62	0.000	15.745***	12.80	0.000	-2.070***	-5.21	0.000
Shb	0.060**	2.22	0.027	0.096	0.98	0.330	0.081***	3.00	0.003
Bsr	-0.004	-1.12	0.263	-0.002	-0.21	0.831	0.002	0.08	0.939
Indep	-0.235***	-4.10	0.000	0.231	1.23	0.221	-0.215***	-3.75	0.000
Dual	-0.108**	-2.58	0.010	-0.073	-0.54	0.586	-0.104**	-2.51	0.012
CSR_1	0.208***	3.04	0.002				0.030**	2.46	0.014
CSR_2	-0.068**	-2.58	0.010				-0.046***	-3.32	0.001
CSR_3	0.007	0.69	0.490				0.025**	2.56	0.011
Gov_1				0.277***	3.71	0.000			
Gov_2				0.119**	2.26	0.024			
Gov_3				0.083*	1.84	0.067			
F-Value	20.40***			40.61***			21.17***		
R^2^	0.228			0.380			0.235		

Note: (1) CSR_1, CSR_2, and CSR_3 are the low-, medium-, and high-input strength intervals of *CSR*, respectively; Sub_1, Sub_2, and Sub_3 are the low-, medium-, and high-strength intervals of *Sub*, respectively; (2) ***, **, and * indicate significant correlation in the 1%, 5%, and 10% levels, respectively; (3) *T* is the t value of significance test of the coefficient under setting condition of heteroscedasticity; and (4) the P value is the result obtained by adopting Bootstrap repeated sampling for 300 times.

Model (1) is the dual-threshold model, with CSR being the threshold variable and independent variable, and brand value being the dependent variable. The test result of the threshold effect indicates that obvious dual threshold effect exists in the influence of CSR on brand value. When CSR does not exceed the threshold value of 0.957, the influence of CSR on brand value shows evident facilitation to a certain extent. When the input level of CSR is 0.957–2.479, CSR has an obvious negative influence on brand value. When the threshold value is larger than 2.479, the correlation between them is not evident. Thus, with the increasing input strength of CSR, CSR shows a positively and then a negatively inverted U-type influence on brand value, and obvious characteristics of threshold effect are presented. With marketing and publicity effect, fulfilling social responsibility can enhance the reputation of enterprises and promote brand value. However, with further increasing CSR inputs, other resources for brand value promotion will be occupied, and brand value will decline. Model (2) is the dual-threshold model, with government subsidy being the threshold variable and independent variable, and CSR being the dependent variable. This model shows that when the government subsidy is lower than the first threshold value, the coefficient is 0.277 and a significant correlation exists at the 1% level. When the threshold value is between the first and second thresholds, the positive influence of government subsidy on CSR weakens obviously and lowers from 0.277 to 0.119, and the significance level also decreases. When the government subsidy exceeds the third threshold, the influence coefficient and significance level will further decrease. Thus, with the increasing strength of government subsidy, facilitation with diminishing marginal utility exists between government subsidy and CSR. Given the existence of corporate moral risk, increasing the amount of government subsidy will lead to lazy and rent-seeking behaviors of enterprises, decline in corporate performance, and decreased input resources of CSR. Model (3) is the dual-threshold model, with government subsidy being the threshold variable, CSR being the independent variable, and brand value being the dependent variable. When the level of government subsidy does not exceed the threshold value of 18.184, CSR has an obvious positive influence on brand value. When the level of government subsidy is 18.184–22.803, a negative influence exists, which indicates that the relationship between them converts to a negative influence at the second threshold effect interval. When it is larger than 22.803, the direction of coefficient influence turns to be positive, and significant correlation exists. Therefore, under the condition of government subsidy threshold, the relationship between CSR and brand value has a significant N-type nonlinear effect. This finding is caused by the fact that with the blessing of government subsidies, enterprises can still spare no effort in investing in activities related to promoting brand value in the case of excessive investment in social responsibility to promote a sustainable growth of brand value.

## Conclusions and managerial implications

On the basis of the panel data of A-share listed companies in the ranking list of China’s Top 500 Most Valuable Brands issued by the World Brand Lab in 2012–2018, a complex nonlinear relationship exists among government subsidy, CSR, and brand value. (1) With CSR being the threshold variable, CSR shows a positively and then a negatively inverted U-type influence on brand value, and obvious characteristics of threshold effect exist. (2) With the government subsidy being the threshold variable and with the increasing strength of government subsidy, facilitation with diminishing marginal utility exists between government subsidy and CSR. (3) With the government subsidy being the threshold variable and with the variation of the threshold interval of government subsidy level, the influence of CSR on brand value shows an obvious N-type law.

The theoretical contributions of this study are as follows. (1) This study constructs the research framework of “Government Subsidy—CSR—Brand Value,” revealing the black box of the interaction effect of government subsidy and CSR on brand value from the perspective of integration. Moreover, it expands the research paradigm of the relationship between CSR and brand value. (2) This study improves the traditional linear model and uses panel threshold data model to study the relationship among government subsidy, CSR, and brand value from a nonlinear perspective. With the change in government subsidy intensity, a nonlinear threshold effect and an optimal intensity interval exist, which compensate for the lack of current research focusing on linear relationship. (3) This study resolves the contradiction between government subsidy and CSR, breaking the original linear thinking. Furthermore, it uses the threshold effect model to confirm the complex relationship between the two from a nonlinear perspective.

The practical implications of this study are as follows. First, CSR shows a positively and then a negatively inverted U-type influence on brand value, which implies that managers should crucially consider which development stage their corporation is in when they decide an investment on CSR activities for a higher brand value [52]. In this manner, they can determine the most suitable investment point concerning their CSR activities to promote the sustainable development of their enterprise and maximize their brand value. Second, government subsidy shows a crowding-in effect on the input of CSR. However, the increasing strength of government subsidy will lead to “free riding behavior” of many enterprises, such as using subsidies for nonbrand building, which is contrary to the will of the government; that is, its crowding-in effect on the input of CSR will gradually weaken. Hence, the strength of government subsidy should also be controlled within a rational scope. Otherwise, the validity of its effect will weaken, and government subsidy will be wasted. Third, relevant subsidy policies should be refined, and application conditions and supervision should be strictly implemented in combination with the characteristics of industries and enterprises. Moreover, on the basis of the N-type law between CSR and brand value in different government subsidy threshold ranges, the government should constantly explore and clarify the moderation interval of their subsidy policies to maximize enterprise brand value and create more enterprises with high brand value through efficient government subsidy policies.
